# A pharmacokinetic and safety study of a fixed oral dose of enzastaurin HCl in native Chinese patients with refractory solid tumors and lymphoma

**DOI:** 10.18632/oncotarget.7875

**Published:** 2016-03-03

**Authors:** Xueying Li, Xiaojie Fang, Su Li, Weijing Zhang, Nong Yang, Yimin Cui, He Huang, Ruiqing Cai, Xiaoting Lin, Xiaohong Fu, Huangming Hong, Tongyu Lin

**Affiliations:** ^1^ Department of Medical Oncology, Sun Yat-Sen University Cancer Center, State Key Laboratory of Oncology in Southern China, Collaborative Innovation Center of Cancer Medicine, Guangzhou, China; ^2^ Department of Clinical Trials Research Center, Sun Yat-Sen University Cancer Center, State Key Laboratory of Oncology in Southern China, Collaborative Innovation Center of Cancer Medicine, Guangzhou, China; ^3^ Department of Lymphoma, The 307th Hospital of Chinese People's Liberation Army, Beijing, China; ^4^ Department of Medical Oncology, Hunan Cancer Hospital, Changsha, China; ^5^ Department of Pharmacy, Peking University First Hospital, Beijing, China

**Keywords:** enzastaurin, pharmacokinetics, safety, solid tumors, lymphoma

## Abstract

**Purpose:**

This study was conducted to assess the pharmacokinetics and safety of enzastaurin in native Chinese patients with refractory solid tumors and lymphoma.

**Methods:**

Eligible patients received 500 mg of enzastaurin orally once daily. The pharmacokinetics of enzastaurin and its metabolites were assessed on days 14 to 18. Patients were allowed to continue receiving the agent in a safety extension phase until disease progression or presentation with unacceptable toxicity.

**Results:**

Twenty-five patients received at least 1 dose of enzastaurin, and twenty-one patients completed the pharmacokinetic phase. Fifteen patients entered the safety extension phase. Except for transient, asymptomatic grade 3 QT interval prolongation in one patient who had baseline grade 2 QT prolongation, other adverse events were of grade 1 to 2. The t_1/2,_ C_av, ss,_ and AUC_τ, ss_ for enzastaurin and its primary active metabolite LSN326020 were 14 and 42 h, 1,210 and 907 nmol/L, and 29,100 and 21,800 nmol•h/L, respectively. One patient with relapsed diffuse large B-cell lymphoma achieved a partial response that lasted for 8.1 months.

**Conclusions:**

The pharmacokinetics of enzastaurin in Chinese cancer patients were consistent with those observed in previous studies abroad. Enzastaurin 500 mg daily was well tolerated by Chinese patients. We recommend 500 mg daily as the phase II dose in this population. Its efficacy in lymphoma deserves further investigation.

**Trial Registration:**

ClinicalTrials.gov: NCT01432951

## INTRODUCTION

As one of the isoforms in the protein kinase C (PKC) family, PKC beta (PKC β) is overexpressed in several solid tumors, including colon cancer, breast cancer, and neuroblastoma, as well as in lymphoid malignancies, including refractory diffuse large B-cell lymphoma (DLBCL), chronic lymphocytic leukemia and follicular lymphoma. PKC β promotes tumorigenesis, enhances tumor cell growth and survival and is associated with a worse prognosis for DLBCL [1–4]. It is also a pivotal component in the B-cell receptor and vascular endothelial growth factor (VEGF) signaling pathways, which correlate to B-cell survival and VEGF-induced tumor angiogenesis, respectively [5–8]. In combination with other PKC subtypes, PKC β can activate the phosphoinositide 3-kinase (PI3K)/AKT pathway, increase cell proliferation and inhibit apoptosis [9].

Enzastaurin, an acyclic bisindolylmaleimide, is a potent and selective inhibitor of PKC β. At clinically achieved plasma concentrations, enzastaurin and its metabolites suppress signaling through not only PKC but also the PI3K/AKT pathway. Accordingly, the inhibition of signaling pathways by enzastaurin suppresses proliferation and induces apoptosis in cultured cell lines from human gastric cancer, pancreatic endocrine cancer, multiple myeloma, and lymphoma [10–13]. Oral dosing with enzastaurin to achieve exposure levels similar to that in human clinical studies suppresses VEGF-induced angiogenesis and the growth of human colon cancer and glioblastoma xenografts [14, 15].

The pharmacokinetic (PK) properties, tolerability and maximum tolerated dose (MTD) of enzastaurin have been studied in Western countries and Japan [16, 17]. However, there are no PK data for this agent in Chinese population. We conducted this phase I study to evaluate the safety and PK of enzastaurin and its active metabolites in native Chinese patients with refractory solid tumors and lymphoma. Antitumor efficacy was also investigated in this population.

## RESULTS

### Patient characteristics

Between December 1, 2011 and January 11, 2013, 26 patients with a variety of refractory solid tumors and lymphoma were enrolled from 4 medical centers in China. Twenty-five patients received at least one dose of enzastaurin; 21 patients completed the PK phase, and 15 patients entered the safety extension phase. The baseline patient characteristics are listed in Table 1.

**Table 1:** Patient characteristics and previous therapy (n = 26)

Characteristic	No. of Patients
Gender	
Male	17
Female	9
Age (years)	
Median (range)	50 (32–78)
ECOG performance status	
0	18
1	6
2	2
Tumor type	
Lymphoma	19
Rectal cancer	3
Nasopharyngeal cancer	2
Breast cancer	1
Ovarian cancer	1
Previous treatment for cancer	
Chemotherapy	26
Molecular targeting therapy	15
Endocrine therapy	1
Surgery	11
Radiotherapy	9

### Adverse events

No deaths or other serious adverse events (AEs) occurred during the study. No patients discontinued because of an AE. In the PK phase, 20 patients (80%) reported at least 1 AE, 14 of which were drug related. In the safety extension phase, 8 patients (53.3%) reported at least 1 AE, 5 of which were drug related. The AEs that were reported in more than 1 patient regardless of causality were electrocardiogram (ECG) QT interval prolongation (*n* = 9), neutropenia (*n* = 3), chromaturia (*n* = 3), increased alanine aminotransferase (*n* = 3), abdominal pain (*n* = 3), increased aspartate aminotransferase (*n* = 2), diarrhea (*n* = 2), leukopenia (*n* = 2), and lymphocytopenia (*n* = 2). AEs were generally grade 1 or 2 according to the Common Terminology Criteria for Adverse Events (CTCAE); 7 patients experienced grade 3 AEs, and 1 experienced a grade 4 AE. The grade 3 AEs were neutropenia, thrombocytopenia, upper respiratory tract infection, lung infection, ECG QT interval prolongation, anemia, lymphocytopenia, leukocytosis, and pain in an extremity; of these, only the ECG QT interval prolongation, which occurred in a 70-year-old female, was considered related to the study drug. The patient had grade 2 QT prolongation at the baseline ECG and developed asymptomatic grade 3 QT prolongation at steady state (day 14). The QT prolongation resolved to grade 2 before the scheduled retreatment on day 18 and remained stable until treatment was discontinued due to disease progression. The grade 4 AE was neutropenia, which occurred in one patient with a primary diagnosis of DLBCL during the period of PK blood draws. The patient withdrew from the study after the last sampling time due to enlargement of superficial lymph nodes based on physical examination. Then, we performed lymph node rebiopsy and bone marrow aspiration, which revealed a second malignancy, acute monocytic leukemia. Therefore, the grade 4 neutropenia was considered caused by leukemia. Only drug-related toxicities are listed in Table 2.

**Table 2:** Number of patients with each enzastaurin-related toxicity at each severity grade (n = 25)

Toxicity, maximum grade	grade 0	grade 1	grade 2	grade 3
QT prolongation	21	1	2	1
Abdominal pain	22	3		
Chromaturia	22	3		
Increased ALT	22	3		
Increased AST	23	2		
Leukopenia	23	1	1	
Lymphocytopenia	23	1	1	
Anemia	24	1		
Neutropenia	24	1		
Thrombocytopenia	24	1		
Diarrhea	24		1	
Insomnia	24		1	
Fatigue	24	1		
Nausea	24	1		

ALT, alanine aminotransferase; AST, aspartate aminotransferase.

### Pharmacokinetics

Evaluable steady-state PK data were obtained from 23 patients. The t_1/2_ was not estimated for 2 patients because they did not complete the washout period. Table 3 summarizes the PK parameters for enzastaurin, LSN326020, and total analytes (enzastaurin + LSN326020 + LSN485912 + LSN2406799) following the 500 mg daily dose of enzastaurin.

**Table 3:** Pharmacokinetic profile of enzastaurin, LSN326020, and total analytes (enzastaurin + LSN326020 + LSN485912 + LSN2406799)

Parameter (unit)	Geometric Mean (% CV)		
	Enzastaurin	LSN326020	Total Analytes
N	23	23	23
C_max, ss_ (nmol/L)	2370 (112)	1070 (38.4)	4140 (81.5)
t_max, ss_ (h)^a^	4.00 (2.0–8.17)	5.97 (0.00–24.00)	4.00 (2.0–8.17)
AUC_τ, ss_ (nmol·h/L)	29100 (128)	21800 (45.5)	89000 (91.9)
C_av, ss_ (nmol/L)	1210 (128)	907 (45.5)	2650 (86.1)
CL/F (L/h)	33.3 (128)	NC	NC
t_1/2_ (h)^b^	14.0 (5.82–38.5)^c^	42.0 (21.8–157)^c^	NC
RA(ratio)	1.50 (28.4)^c^	3.09 (35.3)^c^	NC
MR(ratio)	NC	0.749 (80.5)	NC

a Median and range.

b Geometric mean and range.

c *N* = 21.

AUC_τ, ss_, area under the plasma concentration-time curve during 1 dosing interval at the steady state; C_av, ss_, the average drug concentration under steady-state conditions; CL/F, apparent clearance of drug calculated after extravascular administration; C_max, ss_, maximum observed plasma concentration at the steady state; CV, coefficient of variation; MR, metabolite-to-parent ratio; N, number of patients used in the pharmacokinetic analysis; NC, not calculated; RA, accumulation index; t_½_, half-life; t_max, ss_, time of maximum observed plasma concentration at the steady state.

### Response to treatment

**Figure 1:**
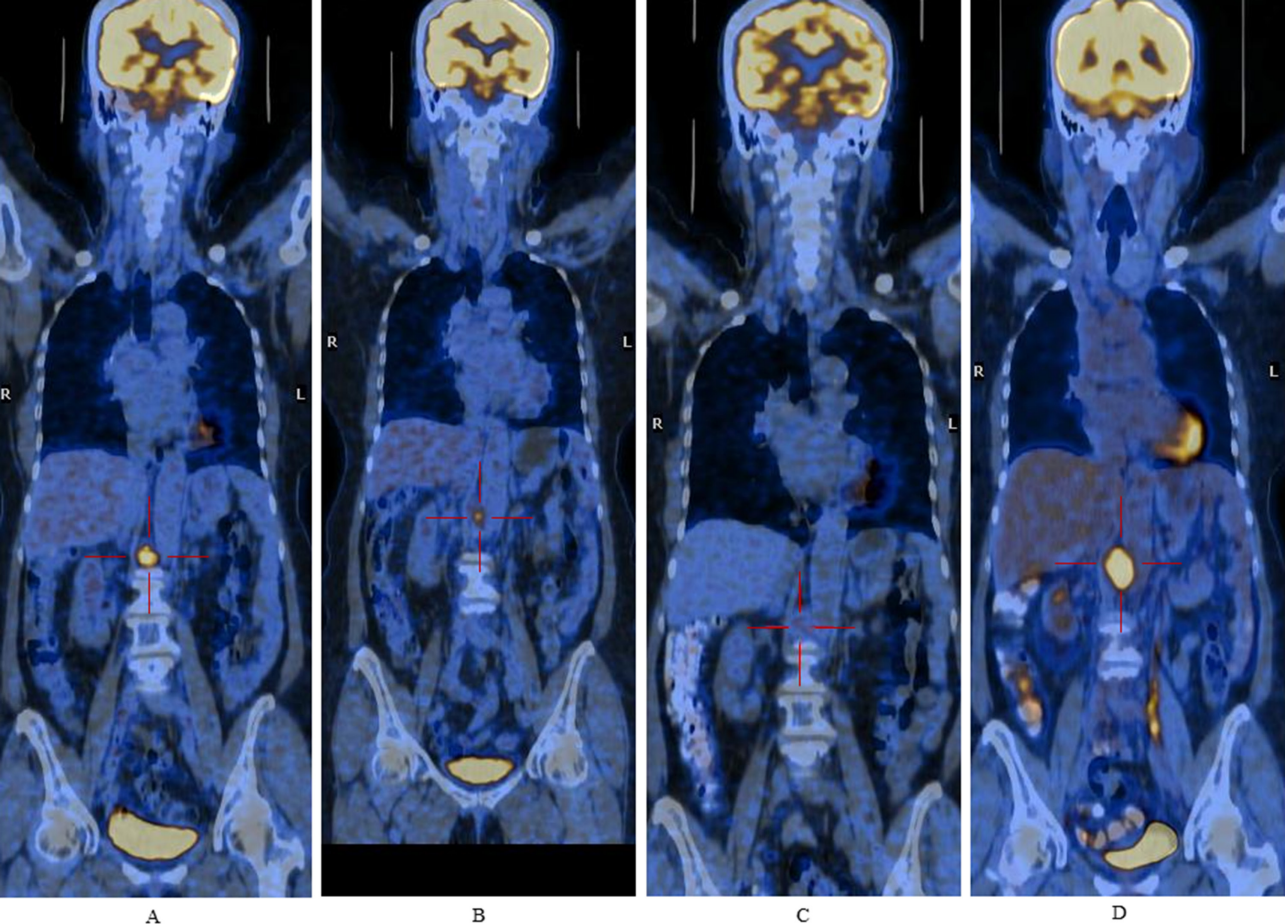
Positron emission computer tomography images demonstrate lymphadenopathy post-crura of the right diaphragm from a relapsed diffuse large B-cell lymphoma patient Figure 1A, Figure 1B, Figure 1C and Figure 1D were images of baseline and after 1.8 months, 6.5 months, 9.9 months of enzastaurin treatment, respectively. A partial response was observed after 1.8 months of treatment and lasted for 8.1 months.

During the safety extension phase, 15 patients received at least one dose of enzastaurin. Disease progression was the major cause of withdrawal from the study. Anti-tumor response was evaluated in 14 patients, including 4 with solid tumors and 10 with lymphoma. One 40-year-old female with relapsed DLBCL who received enzastaurin after a complete response following salvage chemotherapy was disease free for 30 months, and was still using the study drug at the last follow-up on June 18, 2015. A partial response was observed in a 70-year-old female patient with DLBCL whose disease had relapsed after first-line R-CHOP (rituximab, cyclophosphamide, doxorubicin, vincristine and prednisone) therapy and two cycles of salvage chemotherapy with R-GEMOX (rituximab, gemcitabine and oxaliplatin) and R-IMED (rituximab, ifosfamide, methotrexate, etoposide and dexamethasone). The duration of this partial response was 8.1 months (Figure 1). Stable disease was observed in five lymphoma patients, lasting for 22.1 months in one Hodgkin lymphoma (HL) patient and 25.2 months in one follicular lymphoma patient. Four solid tumor patients (two with nasopharyngeal carcinoma, one with rectal cancer, and one with breast cancer) and the remaining three lymphoma patients experienced progressive disease.

## DISCUSSION

This is the first study to evaluate the PK and safety of enzastaurin in native Chinese patients. The agent was administered at a fixed dose of 500 mg once daily, which was recommended as the phase II dose based on PK studies abroad. In this study, enzastaurin was well tolerated at the daily dose of 500 mg. Neither drug-related deaths nor serious AEs were reported. No patient withdrew from the study because of toxicity. Study drug-related AEs were grade 1 to 2, except for QT interval prolongation, which was the only grade 3 AE; this AE occurred in a 70-year-old patient who had baseline grade 2 QT prolongation, and it was transient and asymptomatic. QT prolongation was reported as a dose-limiting toxicity at daily doses of 500 mg or more in previous studies [16–18].

This study demonstrated that the PK of enzastaurin and LSN326020 in Chinese cancer patients appeared to be consistent with data collected from previous studies abroad. Enzastaurin and total plasma analytes achieved a peak concentration within 4 h after one dose. The mean t_1/2_ for enzastaurin and LSN326020 was 14 and 42 h, respectively. Previous studies have shown that the t_1/2_ values of enzastaurin and LSN326020 range from 10.1 to 26.7 h and from 34.8 to 53.9 h, respectively. Steady-state exposures of enzastaurin are approximately 20% higher (RA, 1.19–2.24) than exposures after a single dose, whereas steady-state LSN326020 exposures are approximately two- to four-fold higher (RA, 2.18–3.87) than those after a single dose due to its longer t_1/2_ [16, 17]. In the current study, the RA for enzastaurin and LSN326020 was 1.50 and 3.09, respectively. For a once-daily 500 to 525 mg dose of enzastaurin, the mean steady-state AUC_τ,ss_ values for enzastaurin and LSN326020 have ranged from 23,600 to 44,100 nmol•h/L and from 15,500 to 23,000 nmol•h/L, respectively, in previous studies [16, 19]. The mean AUC_τ,ss_ values for enzastaurin and LSN326020 in the current study were 29,100 and 21, 800 nmol•h/L, respectively, both of which were within the ranges observed in previous studies.

Efficacy was not the primary objective of this study, and it was recorded only for patients who entered the safety extension phase. Progression occurred in all 4 patients with solid cancer, including 2 with nasopharyngeal cancer, 1 with breast cancer and 1 with rectal cancer. The efficacy of single-agent enzastaurin and combination therapy has been investigated in multiple phase II studies of various solid tumors, including malignant glioma as well as breast, ovarian, pancreatic, prostate, colorectal, and non-small cell lung cancer, which have shown poor responses and no survival benefits [20–31]. Of the 10 lymphoma patients, the disease remained stable in 1 HL patient for 22.1 months. Among the remaining 9 patients with non-Hodgkin lymphoma (NHL), 4 achieved stable disease (duration: 25.2 months in 1 follicular lymphoma patient), 1 achieved a partial response (duration: 8.1 months), and 1 experienced a complete response on enzastaurin maintenance therapy after salvage chemotherapy. Single-agent enzastaurin has been studied in relapsed/refractory NHL in phase II trials [32–35]. Although these studies showed limited clinical activity of enzastaurin, select patients achieved relatively long progression-free survival. Similarly, in this study, enzastaurin had limited efficacy against lymphoma. However, a few patients experienced a long progression-free survival period, suggesting that the drug was effective in select patients. The efficacy of enzastaurin as maintenance treatment for prevention of relapse in high risk DLBCL patients was also investigated in a phase III study [36]. In spite of no survival benefit in the unselected population, certain biomarkers might exist that can predict enzastaurin efficacy and identify patient populations that are sensitive to this agent. The possibility of potential predictive biomarkers deserves further investigation to improve the efficacy of enzastaurin against lymphoma.

Overall, the PK profile of enzastaurin in Chinese cancer patients was consistent with that observed in previous studies abroad. Enzastaurin administered at 500 mg orally once daily was well tolerated with minimal toxicity in Chinese patients. We recommend 500 mg daily as the phase II dose in this population. This agent had poor activity in advanced/metastatic solid tumors, but its efficacy in NHL deserves further investigation.

## MATERIALS AND METHODS

### Patient selection

Native Chinese patients with a histologic or cytologic diagnosis of cancer (solid tumor or lymphoma) with clinical or radiologic evidence of locally advanced and/or metastatic disease for which no life-prolonging therapy existed (patients with glioblastoma and other hematologic malignancies [except lymphoma] were excluded) were eligible for this study. Additional inclusion criteria were as follows: 18 years of age and older; an Eastern Cooperative Oncology Group (ECOG) performance status of 2 or less; discontinuation of all previous anti-cancer therapies for at least 30 days prior to study entry (6 weeks for mitomycin-C or nitrosourea), except for luteinizing hormone-releasing hormone analogue therapy for patients with hormone-refractory prostate cancer; adequate bone marrow reserve (absolute neutrophil count ≥ 1.5 × 10^9^/L, platelets ≥ 100 × 10^9^/L, and hemoglobin ≥ 10 g/dL), hepatic function (bilirubin within 1.5 times the upper limit of normal [ULN]; aminotransferase ≤ 2.5 times the ULN or ≤ 5 times the ULN in the presence of liver metastases), and renal function (serum creatinine ≤ 1.5 mg/dL); and an estimated life expectancy that would permit the patient to complete the PK phase and at least 1 cycle of the safety extension phase. Exclusion criteria included central nervous system (CNS) metastases (unless the patient had completed successful local therapy for CNS metastases and had been off corticosteroids for at least 4 weeks before starting the study therapy); serious concomitant systemic disorder; history of human immunodeficiency virus, hepatitis B, or hepatitis C infections; a serious cardiac condition; QTc prolongation > 450/470 ms (male/female) on a baseline ECG; history of a congenital long- QT syndrome; usage of concomitant medications that could prolong the QT/QTc interval; and breast-feeding or pregnant females.

### Study design

This was a non-randomized, open-label, phase I study administering a fixed dose to each consented patient. Eligible patients received 500 mg of enzastaurin once daily. The selected dose was based on two previous phase I trials in advanced cancer, which recommended 525 or 500 mg once daily as the phase II dose [16, 17]. This study complied with all provisions of the Declaration of Helsinki and was conducted in accordance with Good Clinical Practice guidelines. The protocol was approved by the ethical review committee of each participating center. All patients provided written informed consent.

### Drug administration

### Pharmacokinetics phase

Enzastaurin monohydrochloride was supplied by Eli Lilly and Company for oral consumption as 125 mg tablets in bottles. The once-daily 500 mg of enzastaurin was administered approximately 30 min (25 to 45 min) after the start of a meal for 14 continuous days prior to the PK assessments, which occurred from days 14 to 18. Then, the study drug ceased for three days (days 15 to 17) and resumed on day 18, after the last PK sample was obtained. During the study, dosing occurred at approximately the same time of day for each individual patient. Patients were allowed to receive enzastaurin for approximately 2 to 4 weeks after day 18 to assess the potential clinical benefit of continuing treatment in the safety extension phase. Patients who did not enter the safety extension phase underwent washout (stopped the study drug) for approximately 14 days and then withdrew from the study.

### Safety extension phase

Patients who potentially benefitted from continuing treatment were allowed to receive enzastaurin until disease progression or presentation with unacceptable toxicity.

### Patient follow-up and assessments

Eligibility assessments, which consisted of a complete medical history, physical examination, ECOG performance status, vital sign measurements, concomitant drugs, ECG, laboratory tests, one or more relevant radiologic test(s) for tumor measurement (computed tomography scan, magnetic resonance imaging, X-ray, or bone scan) and tumor measurement of palpable or visible lesions, were conducted within 28 days before the lead-in day. Lead-in day (within 7 days before treatment initiation) assessments included physical examination, ECOG performance status, vital sign measurements, concomitant drugs, ECG (at 0 and 4 h) and laboratory tests.

ECGs were performed 4 h after treatment on days 1 and 2. Patients received the same assessments on day 14 as on the lead-in day, except that ECGs were performed predose (0 h) and 4 h postdose. Blood samples for PK were collected from days 14 to 18. After retreatment for 2 to 4 weeks from day 18, patients were re-evaluated to determine whether they should continue in the safety extension phase or washout prior to withdrawal from the study.

All patients who entered the safety extension phase were evaluated with a physical examination, vital signs, laboratory tests, concomitant drugs, ECG at a 4-week follow-up visit, tumor measurements and imaging studies at 4- to 8-week intervals.

### Pharmacokinetic analysis

PK blood samples were taken from day 14 beginning before the enzastaurin dose and ending before the enzastaurin dose on day 18. Nominal collection times were day 14 predose and 1, 2, 4, 6, 8, 10, 24, 48, 72, and 96 h postdose. If a patient missed any of the scheduled enzastaurin doses prior to the PK assessment, the PK assessment was delayed until the patient had received at least 4 consecutive days of enzastaurin doses. The actual sampling time was accurately recorded for analysis. PK blood samples consisted of approximately 2 mL of venous blood to provide approximately 1 mL of plasma for enzastaurin and metabolite analysis. Concentrations of enzastaurin and its metabolites (LSN326020, LSN485912, and LSN2406799) in plasma were analyzed using a liquid chromatography tandem mass spectrometry method validated at a laboratory in China.

Pharmacokinetic parameters were calculated using noncompartmental methods of analysis using WinNonlin (Pharsight Corp., Cary, NC, USA). The maximum plasma concentration (C_max_), time of C_max_ (t_max_), area under the plasma concentration versus time curve during one dosing interval at steady state (AUC_τ, ss_), and average plasma concentration during a dosing interval at steady state (C_av, ss_) were calculated for enzastaurin, its metabolites (LSN326020, LSN485912, and LSN2406799), and total plasma analytes (enzastaurin + LSN326020 + LSN485912 + LSN2406799). The apparent clearance (CL/F) and the half-life (t_1/2_) associated with the terminal rate constant (λ_z_) were calculated for enzastaurin. The t_1/2_ and metabolic ratio (MR) were calculated for LSN326020, LSN485912, and LSN2406799. Total plasma analyte AUC_τ, ss_ and C_av ss_ were calculated by summing the respective AUC_τ, ss_ or C_av, ss_ estimates for each of the 4 individual analytes. Total plasma analyte C_max_ was reported as the highest sum of individual analyte concentrations at any given sampling time during a concentration-time profile, not the sum of the C_max_ of each analyte. A “total” parameter estimate or concentration was calculated if values for enzastaurin and LSN326020 were available; otherwise, “total” was not reported. The AUC was calculated by a combination of linear and logarithmic trapezoidal methods. The linear trapezoidal method was employed up to t_max_, and the log trapezoidal method was then used for data after t_max_. PK measurements were calculated based on the actual collection times. Patients with insufficient data were excluded from the analysis.

### Safety and efficacy evaluations

All observed AEs were graded using the CTCAE, version 3.0 (NCI 2003) [37]. Imaging studies of involved disease sites were repeated every 4 to 8 weeks in patients who entered the safety extension phase. All lesion assessments, whether by physical examination or radiologic methods, were repeated by the same methods at least 4 weeks following the initial observation of an objective response for response confirmation. Responses were recorded based on Response Evaluation Criteria in Solid Tumors, version 1.0 (RECIST 1.0), and Revised Response Criteria for Malignant Lymphoma [38, 39].
